# Treatment-related cardiac complications associated with goal-directed therapy in high-risk surgical patients: a meta-analysis

**DOI:** 10.1186/cc12133

**Published:** 2013-03-19

**Authors:** N Arulkumaran, C Corredor, M Hamilton, M Grounds, J Ball, A Rhodes, M Cecconi

**Affiliations:** 1 St George's Hospital, London, UK

## Introduction

We hypothesized that goal-directed therapy (GDT) is not associated with an increased risk of cardiac complications in high-risk, noncardiac surgical patients. Patients with limited cardiopulmonary reserve are at risk of mortality and morbidity after major surgery [1]. Augmentation of the oxygen delivery index (DO_2_I) with a combination of intravenous fluids and inotropes (GDT) has been shown to reduce the postoperative mortality and morbidity in high-risk patients [2]. However, concerns regarding cardiac complications associated with fluid challenges and inotropes used to augment cardiac output may deter clinicians from instituting early GDT in the very patients who are more likely to benefit.

## Methods

Systematic search of MEDLINE, Embase and CENTRAL databases for randomized controlled trials of GDT in high-risk surgical patients. Studies including cardiac surgery, trauma, and pediatric surgery were excluded to minimize heterogeneity. We reviewed the rates of all cardiac complications, arrhythmias, acute myocardial ischemia, and acute pulmonary edema. Meta-analyses were performed and forest plots drawn using RevMan software. Data are presented as odd ratios (ORs) (95% CIs), and *P *values.

## Results

We identified 23 randomized controlled trials including 2,219 patients, who reported cardiac complications. GDT was associated with a reduction in total cardiovascular complications (OR = 0.55 (0.39 to 0.78), *P *= 0.0007), and with a significantly reduced incidence of arrhythmias (OR = 0.59 (0.38 to 0.91), *P *= 0.02). GDT was not associated with an increase in acute pulmonary edema (OR = 0.68 (0.42 to 1.10), *P *= 0.11) or acute myocardial ischemia (OR = 0.70 (0.38 to 1.27), *P *= 0.23). Subgroup analysis of overall cardiovascular complications revealed that the benefit is most pronounced in patients receiving fluid and inotrope therapy (OR = 0.55 (0.34 to 0.89), *P *= 0.01) to achieve a supranormal oxygen delivery target (OR = 0.50 (0.32 to 0.79), *P *= 0.003), guided by the use of minimally invasive cardiac outmonitoring (OR = 0.50 (0.33 to 0.77), *P *= 0.002).

## Conclusion

Perioperative, physiologically guided, GDT in high-risk surgical patients is not associated and actually reduces postoperative cardiovascular complications.
